# Models and Metrics for Medical Education in Urology: Medical Student Education, Resident Education, and Future Directions

**DOI:** 10.1007/s11934-025-01308-x

**Published:** 2025-12-10

**Authors:** Lakshay Khosla, Kayla M. Keenan, Jay D. Raman

**Affiliations:** 1 Department of Urology, Penn State Health Milton S. Hershey Medical Center, 500 University Drive, C4830, Hershey, PA 17033 USA; 2 College of Medicine, Penn State Health Milton S. Hershey Medical Center, Hershey, PA USA

**Keywords:** Urology education, Medical education, Virtual learning, Surgical simulation training, Artificial intelligence

## Abstract

**Purpose of Review:**

The purpose of this review is to characterize contemporary models and metrics for medical education in Urology.

**Recent Findings:**

Innovative models involve using augmented reality and 3D-printing to enhance stimulation-based training; these are cost-effective and improve surgical skill metrics. Virtual didactics are preferred since they offer flexible self-paced learning. Structured frameworks (e.g., PREDICT, GAPS) increase graduated autonomy without negative impact on patient care. For medical students, Urology interest groups and mandatory skills sessions significantly boost interest and confidence in basic procedures, with targeted outreach helping to address disparities.

**Summary:**

Effective urology education requires standardization, flexibility, and autonomy for residents, and early, equitable exposure for medical students. Integrating evidence-based novel models, supported by technology and Artificial Intelligence for content and feedback, is critical for preparing trainees in the evolving field of Urology. Future research should focus on comparative studies to validate these novel educational models against traditional approaches.

##  Introduction

The landscape of urological resident education is undergoing a profound transformation, moving beyond the traditional “see one, do one, teach one” model to embrace a more structured, competency-based approach. The shift is driven by a confluence of factors, including a rapidly aging population, the increasing complexity of urologic diseases, and the widespread adoption of minimally invasive techniques including, but not limited to, robotic and laparoscopic surgery. For medical students, while the interest of the subspecialty remains steady with as many as 1814 matched urology residents from 2016 to 2021, a decline in formal urology education and clinical exposure remains a challenge. Given this, there are different needs for urological education from the perspective of residents and medical students. Resident Urologic education requires increased standardization, learning flexibility, and improved graduated autonomy, all of which may be achieved by employing new technologies and frameworks. Medical student education in Urology needs further improvement by means of increasing exposure, allowing for focused mentorship, and aiding in development of basic Urologic skills. Many of these needs for residents and medical students can be aided with the use of artificial intelligence, which can streamline creation of content and performance assessments. The purpose of this review is to characterize models and metrics for medical education in Urology.

## Resident Education

Urology education for residents is undergoing a transformation, as new technologies are being utilized to increase standardization and cost-effectiveness while also improving conceptual understanding. In addition, virtual didactics and graduated autonomy are becoming critical components of allowing residents to have efficient learning opportunities that allow them to be better prepared for independent practice.

### Simulation Training Sessions

Residents typically progress from using suture kits and robotic trainers to practicing on animal and cadaver models. Additionally, 87% of program directors agree with the implementation of a standardized simulation curriculum [1]. Despite cadaver labs showing significant improvement in resident surgical skills, they can exceed $10,000 in costs and therefore be difficult to implement at some institutions [1]. To allow for standardization and reduce expenses, newer models are integrating 3D-printing and augmented reality (AR) platforms for surgical preparation. AR-based training has also shown significant improvement in surgical skill metrics such as Global Operative Assessment of Laparoscopic Skills scores and reduces subjective workload for skills training [2]. AR-based models are also less expensive in the long-term, usually requiring a higher upfront cost for hardware followed by lower costs given decreased need for cadaveric material, allowing for remote training, and shortening learning curves. 3D-printing models hold high value for enabling realistic stimulation of both common and rare scenarios across surgical specialties, with trainee satisfaction rates of these models being as high as 95% [3]. 3D-printing models are also cost effective as they usually cost between $100–1000 per unit [4]. Given this, both AR and 3D-printing models have the potential to be effective tools at guiding Urological surgical education.

There is also a lack of standardization of robotic surgical and endourological training. A recent review identified two curricula that may be utilized more universally, the fundamentals of robotic surgery (FRS) and SIMULATE curricula [5]. The FRS employs a modular training model that includes basic instructions for robotic systems, didactic lessons, practical skill stations, team training, and communication exercises. SIMULATE follows progression-focused models that include didactics on semi-rigid and flexible ureteroscopy, virtual reality URS simulation, and fresh-frozen cadaver training. Use of these models have demonstrated significant improvement in performance and a higher percentage of completed procedures, including robotic knot tying, suturing, dissection and vessel coagulation (*p* < 0.01) [6]. Additionally, an improvement in basic TURP skills from 10% to 75% pre- and post- curriculum has been observed. Both these types of curricula which integrate simulation-based training and proficiency-based progression yield superior outcomes when compared to traditional training methods [5]. Despite its effectiveness, proficiency-based progression models remain underutilized in surgical education, with only 67% and 33% of robotic and endourological training tools, respectively, shown to include the models [5].

To allow for a standardized approach to a stimulation curriculum using low-cost material, newer technologies involving 3D-printing and AR need to be further explored and refined. Further research needs to focus on how these newer models, especially when employed under established models such as FRS and SIMULATE, compare against the traditional approaches of cadaver labs.

### Virtual Didactics

Virtual lectures became increasingly popular following COVID-19 and have been the primary method of learning for many trainees. In fact, virtual lectures in surgical training are as effective as in-person lectures with a meta-analysis showing no significant difference in skill-proficiency between the two groups [7]. Urologic trainees actually prefer virtual learning compared to in-person lectures (67.9% vs. 32.1%, *p* < 0.01) [8]. They also prefer case-based learning simulations over traditional lecture formats (58.9% vs. 41.2%, *p* = 0.03) and instruction delivered by national experts compared to in-house faculty (64.3% vs. 35.7%, *p* < 0.01) [8]. Lastly, there is continued preference for a nationally standardized curriculum over a locally designed curriculum (61.8% vs. 38.2%, *p* < 0.01) [8].

There is also increased flexibility in designing education models when utilizing virtual didactics. For example, mandatory topics may be assigned to residents for review to allow for a more directed approach or residents may be allowed to self-select lectures to review to allow for learning autonomy. The value of self-selecting learning topics increases as the postgraduate year increases. For example, fellows showed higher preference for self-selection when compared to junior residents (24.1% vs. 7.3%, *p* < 0.01), who preferred more directed learning [8]. Virtual didactics may allow for a practical approach as trainees can have learning tailored to their comfort level.

Further studies have demonstrated that residents prefer flexibility of choosing specific topics, reviewing material at their own pace, and using virtual platforms. Residents who engage with virtual didactics tend to perform better on exams compared to those who don’t (post test score of 60% vs. 44%, *p* < 0.01) [9]. In fact, residents who viewed 3–5 virtual lectures scored higher on a knowledge-based test than those who watched fewer lectures (*p* < 0.001–0.04.001.04) [9]. Given the ability of virtual learning to allow for flexibility, standardization, and self-paced learning, it has become an increasingly popular tool to incorporate into teaching models in Urological education.

### Graduated Autonomy

There continues to be differing opinions on the degree of surgical autonomy offered to residents as it may be dependent on specific inter-personal relationships, case complexity, and resident experience. There is often a complex balance between optimizing patient care and allowing the learner to gain experience in surgical cases. Studies among surgical residents show that being female, single or childless is associated with lower confidence in operative skills. For urology residents, studies report a 44% decrease in all urology cases completed autonomously by residents [10]. This is observed in various surgeries, including endoscopic and scrotal procedures. In fact, 64% of chief residents reported that they would be unable to perform a robotic partial nephrectomy independently and 61% expressed a lack of confidence in performing a robotic radical prostatectomy [10].

Interestingly, only 7.7% of surgical residents felt confident performing common procedures independently, and these individuals had completed significantly more unassisted cases [11]. Residents with low self-confidence in their surgical skills were more likely to pursue fellowship training. Of note, a survey of fellowship directors found that 66% of incoming fellows were not prepared to operate unsupervised for 30 min [12]. Given this, there is a continued need for models that allow for graduated surgical autonomy for Urological residents.

One effective strategy employs educational time-outs prior to surgery using the PREDICT model [13]. This model requires discussion of the following elements prior to surgery: P-presentation of surgical problems, R-review of operative plan, E-expectations for trainee involvement, D-discussion of patient’s unique considerations, I-incision proposed by trainee, C-concerns anticipated and contingency plans, and T-treatment plan. Additional models employing similar themes have also been suggested such as the Goals, Autonomy, Preparation, and Strategy (GAPS) framework to allow for increased standardization of preoperative briefing and optimizing resident surgical autonomy [14]. These models allow for the teacher to clearly identify learning needs and goals for the trainee and therefore allow for trust in performing portions of the surgical case. Impacts of these structured perioperative dialogue models led to an 88% improvement in resident performance in areas such as goal setting, autonomy, and confidence. It is hypothesized that these debriefs create opportunities to identify and emphasize areas for improvement. Another innovative approach involved a one-month rotation during which chief residents began cases independently using their own operative block time. Attendings supervised the critical portions of each case and assisted as needed [15]. When the outcomes were compared between cases performed independently by chief residents and those performed under a more standard approach, there were no significant differences in 30-day postoperative complications (5.65% vs. 4%, *p* = 0.59) or readmissions (3.7% vs. 3.8%, *p* = 1.00) [15].Resident autonomy may extend past the operative room as a large part of Urological training involves outpatient clinical practice. One approach employed in primary care specialties is to allow for a resident-run clinic where residents see patients, develop plans, and have their own patient panel with faculty available to review care plan. This approach is often shown to allow for easier transition to independent practice. One study showed that resident-run clinics in Urology had as high as 100% patients indicating that they would recommend the clinic to family and friends with the following clinic attributes highly regarded: treatment plans, wait time to obtain appointment, wait time at the clinic, clinic environment, and appointment duration [16]. In fact, 60% of the residents noted that this clinic structure served as a useful tool to help ease the transition to independent practice and faculty evaluations of resident treatment plans and clinic management were overall positive [16].

### Overall Resident Education Model

Overall, there exists a need for increased standardization of resident learning, flexibility of learning options available, and allowing for sufficient graduated autonomy to allow for independent practice following training. Simulation sessions using AR and 3D-printing when employed with established progression-focused curricula such as FRS and SIMULATE can offer a model that allows for increased standardization while optimizing learning efficiency. Additionally, virtual didactic learning increases flexibility, learning autonomy, and ability to pace learning while handling busy clinical responsibilities. Graduated autonomy through PREDICT or GAPS frameworks and utilizing unique clinical opportunities such as independent one-month surgical rotation for chief-residents or resident-run clinics may allow for training that allows for residents to be prepared for independent practice. Further research is still needed to compare outcomes of resident education models that employ these novels against traditional models. Nevertheless, there continues to be potential for growth from these novel approaches in the rapidly advancing world of Urological surgical training.

## Medical Student Education

Many medical schools do not require or offer urology rotations, resulting in lack of essential specialty specific knowledge and skills. This limited exposure not only hinders clinical competency but also reduces early interest in this competitive specialty. The impact of urology interest groups, skill sessions, and virtual education remain an area of interest to enhance exposure to the field.

### Urology Interest Groups

Urology Interest Groups (UIGs) are student led initiatives designed to bring together individuals with shared career interests. While the structure and activities of UIGs vary across institutions, they often include case conferences, journal clubs, career panels, post-match discussions, research opportunities, and access to mentorship. They can also provide exposure to the specialty, as early as the first year of medical school. UIGs serve as an important access point to the field, as only 17% of schools offer required urology clerkship experiences and only 51% offer preclinical urology lectures [17].

Institutions with active UIGs have been associated with a 78.6% increase in student interest in urology (RR 1.3, *p* < 0.05) [18]. Additionally, data shows that 77% of students who participate in subspecialty interest groups match that specialty. This may be attributed to the fact that interests’ groups are associated with 96.3% increased understanding surgical training pathways [18]. UIGs help address the lack of urology exposure by providing early and meaningful experiences with a focus on both the clinical aspects of urology but also on the profession.

Exposure to the specialty is even more of a challenge to medical students without home urology programs. This lack of exposure can also negatively impact match outcomes, as familiarity with programs is linked to higher match rates. One study showed that 75% of applicants matched at an institution they had a prior connection with [19]. To address this barrier, the American Urological Association (AUA) has launched the Future Urology Mentorship Program and UpSquad platform to provide students with in-person and virtual mentorship opportunities. Surveys show that students without home programs valued the “sense of community” that these programs fostered [20].

### Mandatory Clinical Skills Sessions

Another consequence of limited exposure to urology during medical school is the lack of foundational skills in performing urological physical exams and skills that are essential for both medical students and early career residents. To address this gap, some programs have implemented mandatory skills sessions emphasizing hands-on clinical training.

One effective approach involves a combination of didactic and simulation-based learning. In one study, the didactic component was led by practicing urologists with the goal of providing a high-level overview of Urology career paths, while the hands-on sessions focused on clinical skills such as urinary catheter placement, pelvic examinations, and utility of common urologic equipment. Following the training, participants demonstrated an average 37% increase in their understanding of the urology field [21]. Notably, confidence in performing bedside urologic procedures, including catheter placement and physical exams, increased from under 10% to 98–100% [21].

Similar outcomes were observed when a skills-specific curriculum was introduced to third-year medical students prior to entering clerkships to improve bedside readiness, with a focus on catheter placement and bladder irrigation. Students who participated in simulation training demonstrated marked improvements in procedural accuracy, increasing from 66% to 81% (*p* < 0.0001) [18].

Simulation based training that integrates both didactics and hands-on practice serves a dual purpose, specifically providing foundational knowledge of urological principles and offering practical skills that directly enhance student competence.

### Virtual Urology Courses

For students pursuing urology, a competitive specialty, that requires strong USMLE scores, research experience, and exceptional performance on sub-internships, preparation can be challenging. This is due to lack of urological foundational knowledge not adequately addressed during pre-clinical or clinical training. To bridge this gap, a virtual 4-week urology course was developed specifically for sub-interns. The course aimed to build a strong foundation in core urologic topics commonly encountered in clinical practice. At seven-month follow-up, participants demonstrated significantly increased confidence and knowledge in key areas such as hematuria, bladder cancer, kidney stones, prostate cancer, urinary incontinence, and erectile dysfunction (*p* < 0.001) [22]. Additionally, there were marked improvements in essential educational competencies, including patient evaluation, understanding of pathophysiology, critical appraisal of literature, and patient counseling (*p* < 0.001) [22]. This virtual course proved to be an effective tool for preparing students for their urology rotations, not only enhancing knowledge but also facilitating long-term retention and application of clinical skills. The AUA’s core curriculum for medical students also offers a comprehensive set of resources designed to serve as a foundational educational tool in urology. It provides medical students with access to high yield urological principles and concepts, providing access to information they may not have otherwise received through their institution. In addition to the educational content, medical students also have access to information related to the residency application process [23].

Overall, virtual courses can effectively distribute educational content and enhance clinical competency, allowing for long-term confidence, knowledge, and preparedness for urology rotations.

### Focused Mentorship Programs for Underrepresented Medical Students

In addition to the already limited exposure to urology for all medical students, it is also important to address the documented racial and ethnic disparities that exist. Students from underrepresented backgrounds are less likely to engage with UIGs, participate in research, or have access to educational opportunities within the field. Data show that underrepresented students are significantly less likely to attend UIG events (Asian: 23%, Black: 4%, Latinx: 3%, White: 15%; *p* < 0.001) and are more likely to learn about urology through personal or family experiences rather than formal educational channels (Asian: 2%, Black: 19%, Latinx: 6%, White: 9%; *p* < 0.001) [24].

In response, various programs have been created to offer mentorship, clinical exposure, research opportunities, and professional development for students underrepresented in medicine, with the goal of increasing their exposure to urology and ultimately improving residency match rates. Notably, Urology Unbound developed the R. Frank Jones Urology Interest Group (RFJUIG), which has equipped 79% of its applicants with the tools needed to successfully match into urology [25]. The University of California, San Francisco’s Urology Underrepresented Trainees Entering Residency (UReTER) mentorship program, designed to support Black, Indigenous, and Latinx students, reported a 94% match rate among participants, with 38% stating that UReTER played a significant role in their Match Day success [26]. Similarly, the Michigan Urology Academy offers both virtual and in-person training opportunities for medical students at all levels, with a focus on diversifying the future urology workforce. Data show that fourth-year students who participated in the program had an 80.2% match rate, higher than the national average of 71.4% (*p* = 0.0486). Participants also noted feelings of “community support within urology,” as well as the importance of representation and transparency in the application process [27]. Comparable sentiments were reported by students involved in the Future Urology Mentorship Program, who cited a strengthened “sense of community,” particularly benefiting students without a home urology program [20]. Overall, these pipeline initiatives have demonstrated a positive impact on students’ match success and exposure to the field, especially among those who are underrepresented in medicine.

Despite these documented disparities, there is limited research focused on strategies to improve exposure to urology for underrepresented groups. This gap deserves more attention, not only to provide equitable access to career opportunities but also to help develop a more diverse urologic workforce.

### Overall Model for Medical Students

To address potential future workforce shortages, prepare medical students for matching into a competitive specialty, and provide clinical skills, medical school curricula should place greater emphasis on increasing exposure to urology during both the pre-clinical and clinical years. Several strategies have been successfully implemented, including the use of UIGs and mixed-model skills sessions. Mentorship opportunities have proven especially valuable, particularly for students without a home program and those who are underrepresented in medicine. Research indicates that mentorship not only fosters a sense of community and helps demystify the field of urology but may also contribute to improved match rates. These approaches increase knowledge of urology as a career and enhance clinical competencies. These efforts must also advocate for underrepresented student participation, as these individuals often face greater barriers to urological exposure. This can be achieved through integration of urology content into the core curriculum, and inclusion of skills-based training sessions that ensure all students receive equitable exposure to the field.

## Future Directions: the Role of Artificial Intelligence

As artificial intelligence (AI) advances, its role in medical education, particularly for students and residents, is being explored. AI has been proposed as a tool to streamline the creation of educational content and assessments, though concerns remain about the quality of such materials.

### Artificial Intelligence: Creation of Exam Content

One study evaluated AI generated urology exam questions using ChatGPT and Gemini. Experts reviewed the questions, deeming 33.3% as appropriate for use (κ = 0.72, 95% CI: 0.65–0.79). Retention rates by sub-topic ranged from 28.9% (transplantation) to 38.2% (uro-oncology) [28]. When administered to students, the questions led to significant performance improvements, with 95% of participants showing gains between assessments (*p* < 0.001) [28]. While expert oversight remains essential, AI can reduce the time required to develop educational materials and has already demonstrated potential to enhance learning outcomes in urology education.

### Artificial Intelligence: A Tool for Learning Support

AI has also been proposed as a tool to enhance curriculum design by assessing student learning and the effectiveness of educational tools. A systematic review found that AI in medical education is most applied in three domains: learning support, assessment of learning, and curriculum review [29].

In the area of learning support, AI has been used to help students identify knowledge gaps by providing detailed, personalized feedback. For example, in a study involving pathology students, AI analyzed slide-viewing behaviors and exam performance. While AI was able to predict students’ viewing patterns and behaviors, its ability to accurately predict exam answers was limited. More specifically, AI was able to determine that students correctly identifying pathology slides spent less time viewing the slides, were able to quickly go through the view fields, and focus more on areas of diagnostic interest [30].

This advancement is also beginning to influence clinical practice, as AI and machine learning have contributed to the pathological differentiation between benign and malignant tumors, automated Gleason grading, and the prediction of clinical prognosis and molecular subtypes in prostate cancer. Notably, the identification of prostate cancer on pathology slides by board-certified pathologists improved from 70% to 90% with the assistance of AI [31].

### Artificial Intelligence: Performance Based Learning

AI models are also being utilized to allow for performance-based training and real-time feedback on surgical simulation models. In one study, AI-based VR headsets were used to train surgeons to clamp the renal artery on a partial nephrectomy phantom, leading to accuracy as high as 99.91% [32]. Other studies have shown that AI can enhance the safety of robotic surgeries by applying AI models to intraoperative video feeds and instrument kinematics data, enabling automated assessments of surgical skills. For example, deep learning models have shown a mean sensitivity score of 3.52/4.00 for identifying loose connective tissue fibers to create a safe dissection plane during robotic surgery [33]. AI-augmented learning can allow for ease of creating learning models that can lead to increased retention rates of didactic and surgical skill learning. However, further research is needed to validate the accuracy and safety of these tools. Importantly, the integration of AI in healthcare raises ethical concerns, particularly regarding patient safety and its potential impact on both clinical outcomes and surgical education.

### Overall Potential of Artificial Intelligence

Artificial intelligence is becoming increasingly integrated into medicine and has significant potential as a valuable tool in medical education for both medical students and urology residents. Current research shows that AI can support the creation of exam questions, enhance student performance through personalized learning feedback, and assist with curriculum evaluation. These benefits extend into surgical education, where AI-driven virtual reality and real-time skills assessment have achieved high levels of surgical accuracy, particularly in the robotic surgery space. However, continued validation and careful attention to ethical and safety considerations are necessary as AI becomes more widely incorporated into urology education and clinical practice.

##  Conclusion

Successful urology education models focus on improving simulation sessions with progression-focused curricula, developing virtual didactic learning, and increasing opportunities for graduated autonomy through novel frameworks or independent rotations. For medical students, the focus of the education is on early career exposure, basic skill learning, and increasing underrepresented student participation through UIGs and mixed-model skills sessions. AI serves as a novel tool to assist the educator in efficiently developing learning models and the trainee in improving retention of concepts and skills. Further development of Urological models employing the characteristics outlined in this review is encouraged as the landscape of Urological training and surgeries continues to evolve.

## Data Availability

No datasets were generated or analysed during the current study.
